# Uropathogenic *Escherichia coli* infection: innate immune disorder, bladder damage, and Tailin Fang II

**DOI:** 10.3389/fcimb.2024.1322119

**Published:** 2024-04-04

**Authors:** Zong-ping Li, Jun Li, Tong-lu Li, Zhi-yong Song, Xue-zhong Gong

**Affiliations:** Department of Nephrology, Shanghai Municipal Hospital of Traditional Chinese Medicine, Shanghai University of Traditional Chinese Medicine, Shanghai, China

**Keywords:** uropathogenic Escherichia coli, urinary tract infection, innate immunity, bacterial escape, Chinese herbal drugs

## Abstract

**Background:**

Uropathogenic *Escherichia coli* (UPEC) activates innate immune response upon invading the urinary tract, whereas UPEC can also enter bladder epithelial cells (BECs) through interactions with fusiform vesicles on cell surfaces and subsequently escape from the vesicles into the cytoplasm to establish intracellular bacterial communities, finally evading the host immune system and leading to recurrent urinary tract infection (RUTI). Tailin Fang II (TLF-II) is a Chinese herbal formulation composed of botanicals that has been clinically proven to be effective in treating urinary tract infection (UTI). However, the underlying therapeutic mechanisms remain poorly understood.

**Methods:**

Network pharmacology analysis of TLF-II was conducted. Female Balb/C mice were transurethrally inoculated with UPEC CFT073 strain to establish the UTI mouse model. Levofloxacin was used as a positive control. Mice were randomly divided into four groups: negative control, UTI, TLF-II, and levofloxacin. Histopathological changes in bladder tissues were assessed by evaluating the bladder organ index and performing hematoxylin-eosin staining. The bacterial load in the bladder tissue and urine sample of mice was quantified. Activation of the TLR4-NF-κB pathway was investigated through immunohistochemistry and western blotting. The urinary levels of interleukin (IL)-1β and IL-6 and urine leukocyte counts were monitored. We also determined the protein expressions of markers associated with fusiform vesicles, Rab27b and Galectin-3, and levels of the phosphate transporter protein SLC20A1. Subsequently, the co-localization of Rab27b and SLC20A1 with CFT073 was examined using confocal fluorescence microscopy.

**Results:**

Data of network pharmacology analysis suggested that TLF-II could against UTI through multiple targets and pathways associated with innate immunity and inflammation. Additionally, TLF-II significantly attenuated UPEC-induced bladder injury and reduced the bladder bacterial load. Meanwhile, TLF-II inhibited the expression of TLR4 and NF-κB on BECs and decreased the urine levels of IL-1β and IL-6 and urine leukocyte counts. TLF-II reduced SLC20A1 and Galectin-3 expressions and increased Rab27b expression. The co-localization of SLC20A1 and Rab27b with CFT073 was significantly reduced in the TLF-II group.

**Conclusion:**

Collectively, innate immunity and bacterial escape from fusiform vesicles play important roles in UPEC-induced bladder infections. Our findings suggest that TLF-II combats UPEC-induced bladder infections by effectively mitigating bladder inflammation and preventing bacterial escape from fusiform vesicles into the cytoplasm. The findings suggest that TLF-II is a promising option for treating UTI and reducing its recurrence.

## 1 Introduction

Urinary tract infection (UTI) is one of the most prevalent bacterial infections globally, afflicting approximately 150 million individuals each year (Tamadonfar et al., 2019). Approximately 60% of women will encounter UTI at least once in their lifetime, with 30%-40% experiencing recurrent UTI (RUTI) (Kwok et al., 2022). Uropathogenic *Escherichia coli* (UPEC) is the culprit behind over 80% of UTI (Ronald, 2003), and interestingly, the original strain accounts for 68% of recurrent infections (Bruxvoort et al., 2020). The virulence genes of UPEC strains are extensive and significantly diverse, resulting in complex and varied symptoms in individuals with UTI. DNA microarray probes can provide accurate diagnosis and definitive treatment for UTI caused by UPEC pathotypes (Behzadi and Behzadi, 2017). However, despite administering appropriate antibiotic therapy, UTI has progressively become more treatment-resistant, a trend attributed to the increase in antibiotic resistance (Tamadonfar et al., 2019). The prevalence of RUTI severely compromises patients’ quality of life and poses a remarkable economic burden on healthcare systems. Consequently, there is an urgent need for further research to advance treatment options.

The urinary tract is an exceedingly challenging environment for pathogen infection, largely attributed to the robust innate immune defense system (Song et al., 2007b; Behzadi et al., 2023). TLRs are potential targets for therapeutic intervention in inflammation-related diseases, autoimmune diseases, and microbial infections. TLRs act as innate immune receptors that selectively bind to pathogenic ligands of pathogen-associated molecular patterns, triggering an innate immune response through activation of inflammatory signaling cascades (Mukherjee et al., 2019; Mukherjee et al., 2023). Toll-like receptor 4 (TLR4), expressed on bladder epithelial cells (BECs), primarily recognizes lipopolysaccharide (LPS) on the surface of gram-negative bacteria (Akira et al., 2006). The binding of bacterial LPS to the TLR4-MD2 complex triggers a signaling cascade via MyD88-dependent or non-dependent pathways, culminating in the activation of NF-κB and the expression of immunomodulatory cytokines such as interleukin (IL)-6 and IL-8 (Song et al., 2007a; Patra et al., 2021). While the innate immune response effectively clears extracellular pathogens, UPEC employs a strategy to evade host defenses by colonizing fusiform vesicles on infected BECs and infiltrating the cells to form intracellular bacterial communities (IBCs). The phosphate transporter protein SLC20A1, primarily responsible for phosphate transport into host cell cytoplasm, is expressed in the bladder (Wagner et al., 2014; Rieke et al., 2020). During UPEC infection, SLC20A1 localizes to the membrane of Rab27b+ fusiform vesicles in superficial BECs. Increased SLC20A1 expression facilitates the transfer of phosphate from bacterium-containing vesicles (BCVs) to the cytoplasm (Song et al., 2009; Koumakis et al., 2019). UPEC senses the low phosphate concentration within BCVs, prompting it to escape from the fusiform vesicles and elude host immune mechanisms to establish IBCs (Snijder et al., 1999). Therefore, devising strategies to inhibit UPEC’s entry into the cytoplasm presents a promising approach to UTI treatment and recurrence prevention.

Due to the global increase in antimicrobial resistance, some researchers suggest that “prevention is better than cure”. This means replacing current antibiotic therapies with effective preventive measures, which is a promising alternative (Behzadi et al., 2023). Chinese herbal medicine (CHM) has a rich history spanning more than 2000 years as a traditional intervention for UTI symptoms. It has proven highly effective in symptom alleviation and reducing antibiotic use in cases of UTI (Flower et al., 2019). Tailin Fang II (TLF-II), an empirical prescription, was developed by our research group based on TLF (Li et al., 2022) for the prevention and treatment of UTI. It is composed of some herbs such as *Pseudostellariae Radix* (Taizishen, TZS), *Rehmanniae Radix* (Shengdihuang, SDH), *Sargentodexae Caulis* (Daxueteng, DXT), *Coicis Semen* (Yiyiren, YYR), *Plantaginis Semen* (Cheqianzi, CQZ), and *Polygoni Cuspidati Rhizoma et Radix* (Huzhang, HZ) etc. and has been frequently employed in the clinical. TLF-II was obtained by decocting method. Animal and clinical studies have demonstrated the effectiveness of TLF, especially in terms of mitigating tubular and interstitial inflammation and inhibiting UTI-induced renal fibrosis (Gong et al., 2006; Gong et al., 2010; Gong et al., 2021). Meanwhile, it could significantly lower the expression levels of renal tubular injury markers (urinary NAG and β2-MG) and pro-fibrotic factors (MCP-1 and TGF-β1) of recurrent UTI patients (Li et al., 2022). However, the regulatory role and underlying mechanisms of TLF-II remain unexplored.

The study was divided into two parts: the dry lab (silico and bioinformatic studies) and the wet lab (*in vivo* studies) (Behzadi and Ranjbar, 2019). The therapeutic potential of TLF-II in a UTI mouse model was investigated based on our network pharmacology results. *In vivo* studies showed that TLF-II inhibited UPEC-induced bladder inflammation and attenuated bladder injury. Its anti-inflammatory effects may be mediated by modulating the TLR4-NF-κB pathway. Subsequently, our data revealed TLF-II’s capacity to inhibit UPEC intrusion into the cytoplasm by reducing SLC20A1 expression. Based on these findings, TLF-II emerges as a promising novel treatment option for UTI.

## 2 Materials and methods

### 2.1 Dry lab

#### 21.1 Ultra-high performance liquid chromatography

An ultra-high performance liquid chromatograph waters ACQUITY (waters, USA) was used to identify components in TLF-II. The sample was injected into a column (100*2.1 mm, 1.8 μm). The HPLC system consisted of 0.1% formic acid aqueous solution (A) and 0.1% formic acid acetonitrile solution (B). The flow rate was maintained at 0.35 mL/min, while the column temperature was set at 45°C. The LC-MS data were processed by the software Progenesis QI V2.3 (Nonlinear, Dynamics, Newcastle, UK) for baseline filtering, peak identification, integral, retention time correction, peak alignment, and normalization. Main parameters of 5 ppm precursor tolerance, and 10 ppm product tolerance.

#### 2.1.2 Network pharmacology

Natural compounds present in TLF-II were retrieved from the Traditional Chinese Medicine (TCM) Systems Pharmacology Database and Analysis Platform (TCMSP) and filtered based on their pharmacokinetic characteristics (oral bioavailability ≥ 30% and drug-likeness ≥ 0.18) (Ru et al., 2014). Simultaneously, the target genes of these components were obtained from TCMSP. In cases where information on the target genes of the components was lacking, the target genes were predicted using PharmMapper (Liu et al., 2010). Target genes related to UTI were sourced from the GeneCards database and DisGeNET databases (Piñero et al., 2020). Duplicate entries were removed, resulting in a compilation of UTI-related target genes. The target genes associated with both TLF-II and UTI were identified as potential targets for drug intervention in the disease. To construct a protein-protein interaction network and identify key gene modules, the potential target genes were imported into the STRING database, and the MCODE plugin in Cytoscape software (version 3.7.1) was utilized (Szklarczyk et al., 2021). Concurrently, Cytoscape was employed to create a network illustrating the complex relationship between TLF-II, its components, and the target genes. The potential target genes were subjected to GO biological process and KEGG pathway enrichment analyses using the Database for Annotation, Visualization, and Integrated Discovery (DAVID) (Huang et al., 2009). Furthermore, transcription factor target enrichment analysis of the potential target genes was conducted using the TRRUST database (Han et al., 2018). Finally, before conducting molecular docking, the natural compounds and target proteins were processed using Discovery Studio v16.

#### 2.1.3 Statistical analysis

Statistical analyses were performed using GraphPad Prism 9.0 software (Paragraph Software, USA). All data were analyzed using one-way analysis of variance. The results were presented as mean ± standard error of the mean. A two-sided P-value of < 0.05 was considered statistically significant.

### 22 Wet lab

#### 2.2.1 TLF-II samples preparation

Botanical drugs of TLF-II were purchased from Shanghai WanShiCheng Pharmaceutical Co. Ltd. of China and identified by the Traditional Chinese Medicine Pharmacy of Shanghai Municipal Hospital of Traditional Chinese Medicine. TLF-II was obtained by decocting method. All plant names were checked by Pharmacopoeia of the People’s Republic of China (https://db.ouryao.com/yd2020/) and the Plant List (http://www.theplantlist.org). The dose of TLF-II (equivalent clinical dose) used in the mouse experiments was 2.28 g/kg according to the body surface area normalization method.

#### 2.2.2 Animals and experimental groups

All animal care and experimental procedures described in this study adhered to the guidelines provided by the Shanghai Hospital of Traditional Chinese Medicine Ethics Committee and international animal welfare standards (Ethics review number: 2023012). The procedures were carried out following the Guide for the Care and Use of Laboratory Animals (National Institutes of Health Publication, USA, No. 85-23, revised 1996). Female Balb/C mice (6-8 weeks old, weighing 19 ± 2 g) were procured from Shanghai Slack Laboratory Animal Co., Ltd. (animal license number: SCXK [Shanghai] 2022-0004). The mice were housed in specific pathogen-free animal care facilities, maintained in an environment-controlled room (23 ± 2°C, 50 ± 10% humidity, and 12h/12h light/dark cycle), and provided a standard chow diet for 7 days as an adaptation period before the experiment.

Following this adaptation period, all mice were randomly divided into four groups: negative control (NC) group (n=10), UTI group (n=10), UTI+TLF-II group (n=10), and UTI+levofloxacin (LVFX) group (n=10). Most mice are naturally resistant to prolonged UTI due to physiological differences between mice and humans (Murray et al., 2021). A therapeutic course of TLF-II is usually for 7 days. In order to investigate the effect of TLF-II on the prevention and treatment of UTI and to minimize the impact of urethral bacterial self-clearance in mice after modeling, we administered a TLF-II decoction (2.28 g/kg/d) to the TLF-II group of mice through gavage for 5 days before modeling (based on the optimal dosing concentration determined in our previous animal studies (Gong et al., 2010)). Subsequently, we administered TLF-II decoction to the mice for 2 days through gavage after 24 hours of successful modeling to ensure that bladders were sampled within 72 hours after modeling when bacterial levels were high. The remaining groups received saline through oral gavage. In the LVFX group, LVFX (50 mg/kg/d) was administered to the mice intraperitoneally for 2 days, 24 hours after modeling, while the mice in the other groups received phosphate-buffered saline (PBS).

#### 2.2.3 Bacterial strains and culture conditions

*E. coli* CFT073 (O6:K2:H1; x0098, BioSCI, China), a prototypical uropathogenic strain causing UTI (Shea et al., 2020; García et al., 2022), was used throughout the study. CFT073 was activated and cultured at 37°C, and the bacteria were collected by centrifugation at an optical density of approximately 0.8. The bacteria were resuspended in 10% glycerol and transformed with the pA127Tc-mcherry plasmid by electrostimulation on ice (Tanaka et al., 2022). After transfer to a tetracycline (TC)-resistant Luria-Bertani (LB) plate, red strains were selected under a fluorescence microscope and inoculated into a TC-resistant medium (10 μg/mL) to obtain them. The bacterial solution was mixed with sterilized 50% glycerol to a final concentration of 10%-15% and stored at −80°C. Plasmid mapping is depicted in **Supplementary Figure S1**. Bacterial strains were cultured in LB broth (A507002; Sangon Biotech, China) under aerobic conditions at 37°C. The medium was supplemented with TC (10 μg/mL) when needed.

#### 2.2.4 UTI model

The mouse bladder infection model was established following previously described methods with appropriate modifications (Alteri et al., 2009; Hung et al., 2009). Prior to modeling, female Balb/C mice aged 6-8 weeks were deprived of water overnight to prevent UPEC from being flushed out with urine due to reflexive urination. Mice were anesthetized with 1% pentobarbital, and the urethral orifice was disinfected with iodophor. UPEC CFT073 strains suspended in 50 μL of PBS (≥ 2×10^8^ CFU) were transurethrally inoculated. Successful modeling was confirmed by testing urine UPEC levels ≥ 10^4^ CFU/mL. Mice were killed by CO_2_ asphyxiation at the specified time points, and their bladders were removed aseptically.

#### 2.2.5 Bladder morphology and organ index

The organ index is also known as the relative organ weight (Lazic et al., 2020). An increase in organ index indicates that the organ has experienced congestion, edema, or hyperplasia and hypertrophy, and can be used to assess organ damage in a relatively quantitative manner. We followed a previously described method with slight modifications (Chen, 2022). Female Balb/C mice, aged 6-8 weeks, were transurethrally inoculated with 2 × 10^8^ CFU of UPEC CFT073 strains suspended in 50 µL of PBS. After the indicated drug interventions, the weight of each mouse was recorded; the bladder was gently expressed to remove residual urine, after which the bladder was aseptically removed and weighed to calculate its organ index (bladder organ index = bladder weight (mg)/body weight (g) × 100%). Simultaneously, the morphological size and pathological condition of the mice bladders we observed and documented.

#### 2.2.6 Hematoxylin and eosin staining

Bladder tissues were fixed in 4% paraformaldehyde, embedded in paraffin, cut into 5-μm-thick sections, and preheated in an oven at 60°C for 8-10 minutes. Subsequently, the sections were dewaxed sequentially in xylene, anhydrous ethanol, 95% ethanol, and 85% ethanol. Hematoxylin solution was applied to the sections for 3-5 minutes, followed by the application of eosin solution for 2 minutes. The sections were dehydrated sequentially in 95% ethanol and anhydrous ethanol, and neutral gum was used for sealing after xylene permeation. Histomorphological changes were observed under a microscope (DM6B; Leica, Germany).

#### 2.2.7 Urine analysis and inflammation score

To evaluate inflammation severity in mice, we counted and scored polymorphonuclear leukocytes (PMN) in their urine sediment. Mice urine sediments were obtained, and 5 µL of the sediment was smeared onto glass slides, after which the smears were fixed in 95% ethanol for 15 minutes. Papanicolaou staining was performed according to the manufacturer’s instructions (G1614, Solarbio, China). A microscope (DM6B; Leica, Germany) was used to count and score PMN in five random fields of view for each mouse. The scores ranged from 0 to 4, where scores ≤1 were recorded as 0 and scores ≥20 were recorded as 4, as previously described with minor modifications (Joshi et al., 2021). To quantify the bacterial load in urine, fresh urine samples were obtained from mice aseptically before killing. The samples were serially diluted in PBS, and 5 μL of each dilution was added onto LB plates and incubated for 24 hours. Bacterial titers were calculated as log_10_ CFU/mL.

#### 2.2.8 Quantitative analysis of bacteria in the bladder tissue

The aseptically harvested bladder tissues were homogenized in 1 mL of sterile PBS. After centrifuging the homogenate at 300 rpm for a short period, the supernatant was collected. The supernatant was serially diluted, and 5 μL of the serially diluted supernatant was added onto LB plates and incubated for 24 hours, followed by colony counting. Bacterial titers were calculated as log_10_ CFU/mL (Joshi et al., 2021).

#### 2.2.9 Immunohistochemistry

Bladder tissues were fixed with 4% paraformaldehyde, embedded in paraffin, and cut into 5-μm-thick sections. Antigen retrieval was performed with trypsin at 37°C. The primary antibodies (rabbit anti-TLR4, AF7017, Affinity, China; rabbit anti-SLC20A1, A03537-1, Boster, China) were incubated overnight at 4°C. After washing with PBS three times, the tissues were incubated with an enhancer and the secondary antibodies (PV-9001, ZSGB-BIO, China) were incubated separately at 37°C for 20 minutes. After color development with DAB, hematoxylin solution was applied for 2-3 minutes. Dehydration with 95% ethanol and anhydrous ethanol was performed sequentially, and neutral gel was applied after xylene permeation. Finally, the results were observed using a microscope (DM6B; Leica, Germany).

#### 2.2.10 ELISA analysis

Blood samples were collected from the ocular orbit of mice and centrifuged at 3000 rpm at 4°C for 5–10 minutes, followed by supernatant collection. ELISA kits (JL20268 and JL18442, Jianglai, China) were used to measure IL-6 and IL-1β levels following the manufacturer’s protocol.

#### 2.2.11 Western blotting

Proteins in the sample were lysed in radioimmunoprecipitation assay lysis buffer at the indicated post-infection time points, and the bicinchoninic acid assay was used for total protein quantification. Subsequently, 20 μg of the protein sample was separated by sodium dodecyl sulfate–polyacrylamide gel electrophoresis, after which the separated proteins were transferred onto polyvinylidene difluoride membranes (Millipore, USA). The membranes were blocked with 5% milk powder for 1 hour at room temperature and then incubated with primary antibodies overnight at 4°C. The primary antibodies used included rabbit anti-NF-κB p65 (8242s, Cell Signaling Technology, USA), rabbit anti-p-NF-κB p65 (3033s, Cell Signaling Technology, USA), rabbit anti-SLC20A1 (A03537-1, BOSTER, China), rabbit anti-Rab27b (DF12060, Affinity, China), rabbit anti-Galectin-3 (orb214186, Biobyt, UK), and GAPDH (AF7021, Affinity, China). The membranes were washed three times with 0.1% Tween 20-PBS and then incubated with suitable secondary antibodies (A0208, Beyotime, China) for 1 hour at room temperature. After three additional washes with 0.1% Tween 20-PBS, the blots were developed using the ChemiDoc™ MP imaging system. GAPDH was used as a loading control. Bio-Rad Image Lab software was used to perform densitometry analysis. Protein quantification was performed using Image J software. Experiments were repeated three times.

#### 2.2.12 Immunofluorescence colocalization

The mCherry-expressing CFT073 strain was transurethrally inoculated into 6 to 8-week-old female Balb/C mice, and bladders were immediately placed in optimal cutting temperature compound (OCT) for embedding. All frozen tissue sections were fixed in 4% paraformaldehyde for 20 minutes, protected from light, washed with PBS, permeabilized in 0.1% Triton X-100 PBS for 10 minutes, and incubated with 5% bovine serum albumin (BSA; WH3044, WellBio, China). Primary antibodies were appropriately diluted with 5% BSA, including mouse anti-Rab27b antibody (66944-1-Ig, Proteintech, China) and rabbit anti-SLC20A1 antibody (12423-1-AP, Proteintech, China). These diluted antibodies were applied to the tissues and incubated overnight at 4°C. Subsequently, Alexa Fluor 488-conjugated (AS053, ABclonal, China) and Alexa Fluor 647-conjugated (AS078, ABclonal, China) secondary antibodies were applied to the tissues for 60 minutes at room temperature. After staining with DAPI (C02-04002, Bioss, China) for 10 minutes at room temperature, the sections were sealed with an autofluorescence burst-blocking solution and observed using a laser scanning confocal microscope (SP8; Leica, Germany).

## 3 Results

### 3.1 Chemical composition of TLF-II

**Figure 1:**
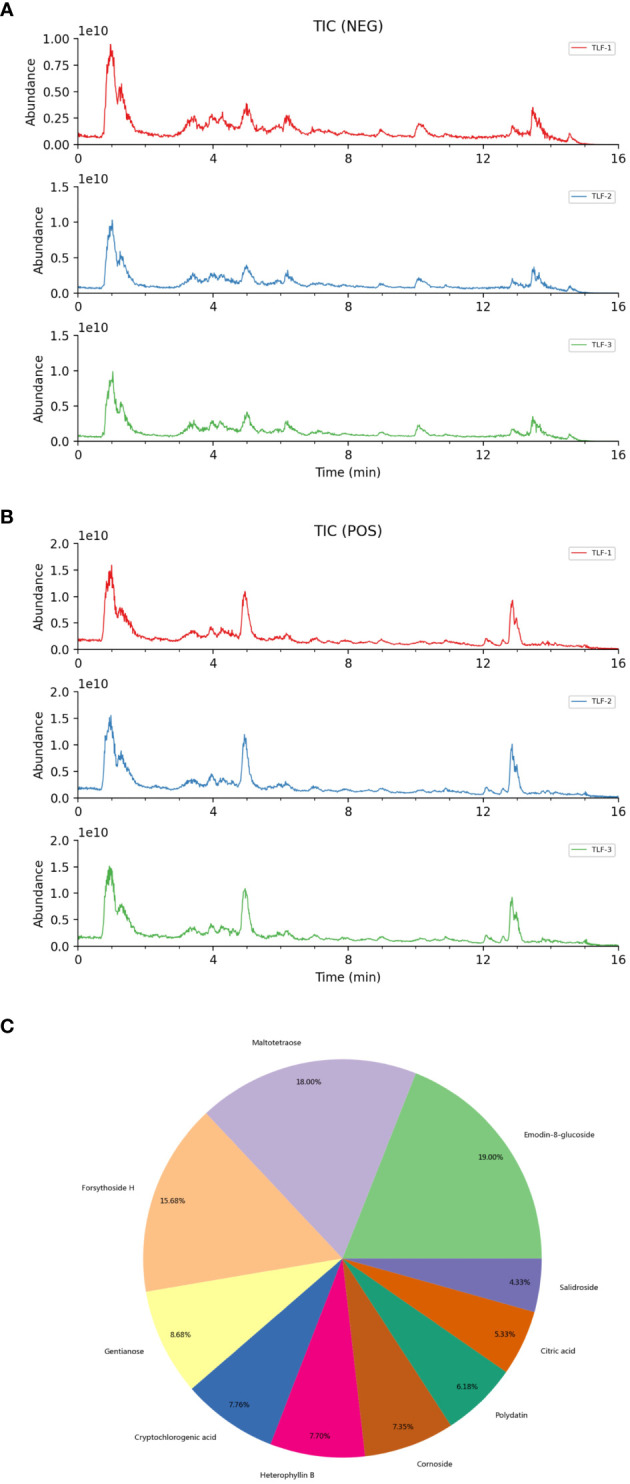
Identification of chemical composition of TLF-II. **(A, B)** Metabolite detection chromatography spectrometry extraction ion diagram. **(C)** The main chemical components of TLF-II.

To identify the chemical constituents in TLF-II, compound identification was performed using LuMet-TCM and the Herb databases based on the precise mass-to-charge ratio (m/z), secondary fragments, and isotopic distribution. **Figures 1A, B** shows the ion extraction (TIC) by chromatography spectrometry for metabolite detection. To ensure the accuracy of the identification results, the EIC plots and secondary mass spectra with secondary fragment structure annotations, labeled the fragmentation information and annotated the fragmentation structure (**Supplementary Figure S2**). **Figure 1C** summarizes the main chemical components of TLF-II, including Emodin-8-glucoside, Maltotetraose, Forsythoside H, Gentianose, Cryptochlorogenic acid, etc. For all identified components, pie charts were plotted according to their quantity and content under each chemical classification category (**Supplementary Figure S3**). **Supplementary Table S1** shows detailed information on all target metabolites of TLF-II.

### 3.2 Potential effects and targets of TLF-II on UTI by network pharmacology

**Figure 2:**
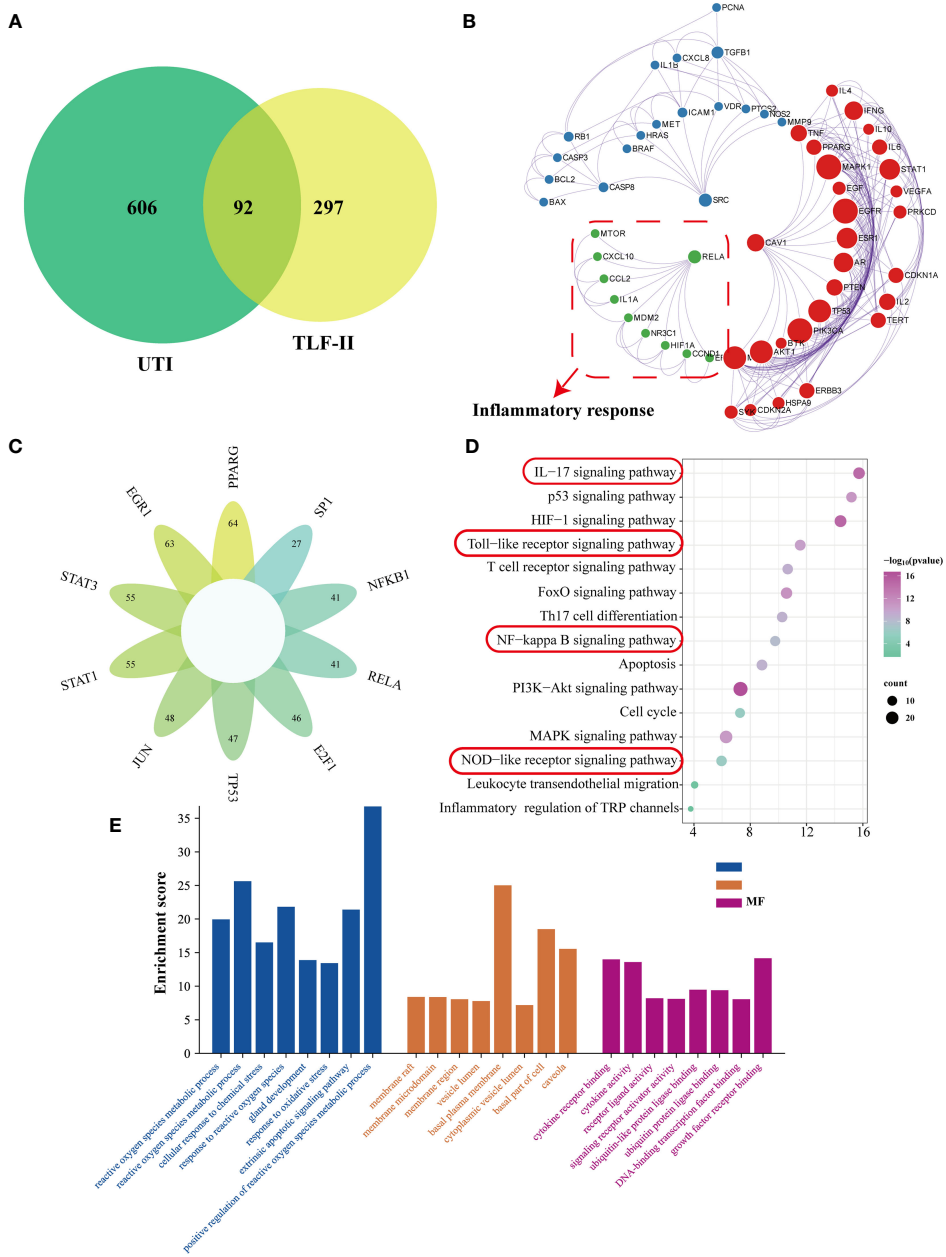
Network pharmacological analysis of the TLF-II intervention for UTI. **(A)** Venn diagram illustrating the intersection of drugs and disease genes. **(B)** Screening of MCODE gene modules based on the protein-protein interaction (PPI) network. **(C)** Enrichment analysis of transcription factor targets. **(D)** KEGG pathway enrichment analysis. **(E)** GO biological process enrichment analysis.

The study screened the GeneCards database to identify UTI-related genes (relevance score ≥25), which yielded 544 target genes. Combining and de-emphasizing these gene lists resulted in a total of 698 UTI-related target genes. This study then focused on TLF-II and identified 398 target genes associated with TLF-II from the TCMSP database. **Figure 2A** illustrates the intersection, revealing 92 potential target genes for pharmacological intervention in UTI, targeted by 31 natural compounds (**Supplementary Table S1**). As shown in **Figure 2B**, the MCODE algorithm revealed three core gene modules among these potential targets, with the green module demonstrating a closer relationship with the inflammatory response. Transcription factor enrichment analysis indicated that these genes were predominantly regulated by PPARG, EGR1, STAT3, STAT1, JUN, TP53, E2F1, RELA, NFKB1, and SP1 (**Figure 2C**). KEGG enrichment analysis shown in **Figure 2D** highlighted multiple immune- and inflammation-related pathways, including the IL-17 signaling pathway, Toll-like receptor signaling pathway, NF-κB signaling pathway, and NOD-like receptor signaling pathway. GO enrichment analysis led to further categorization, namely biological process (BP), cellular component (CC), and molecular function (MF). The top 8 enriched gene biological function catalogs in each category were represented in histograms (**Figure 2E**). The BPs involved in these genes encompassed processes such as reactive oxygen species metabolic processes, regulation of reactive oxygen species metabolic processes, cellular response to chemical stress, and response to reactive oxygen species. CC mainly included membrane raft, membrane microdomain, membrane region, and vesicle lumen. MF covered functions such as cytokine receptor binding, cytokine activity, receptor-ligand activity, and signaling receptor activator activity.

**Figure 3:**
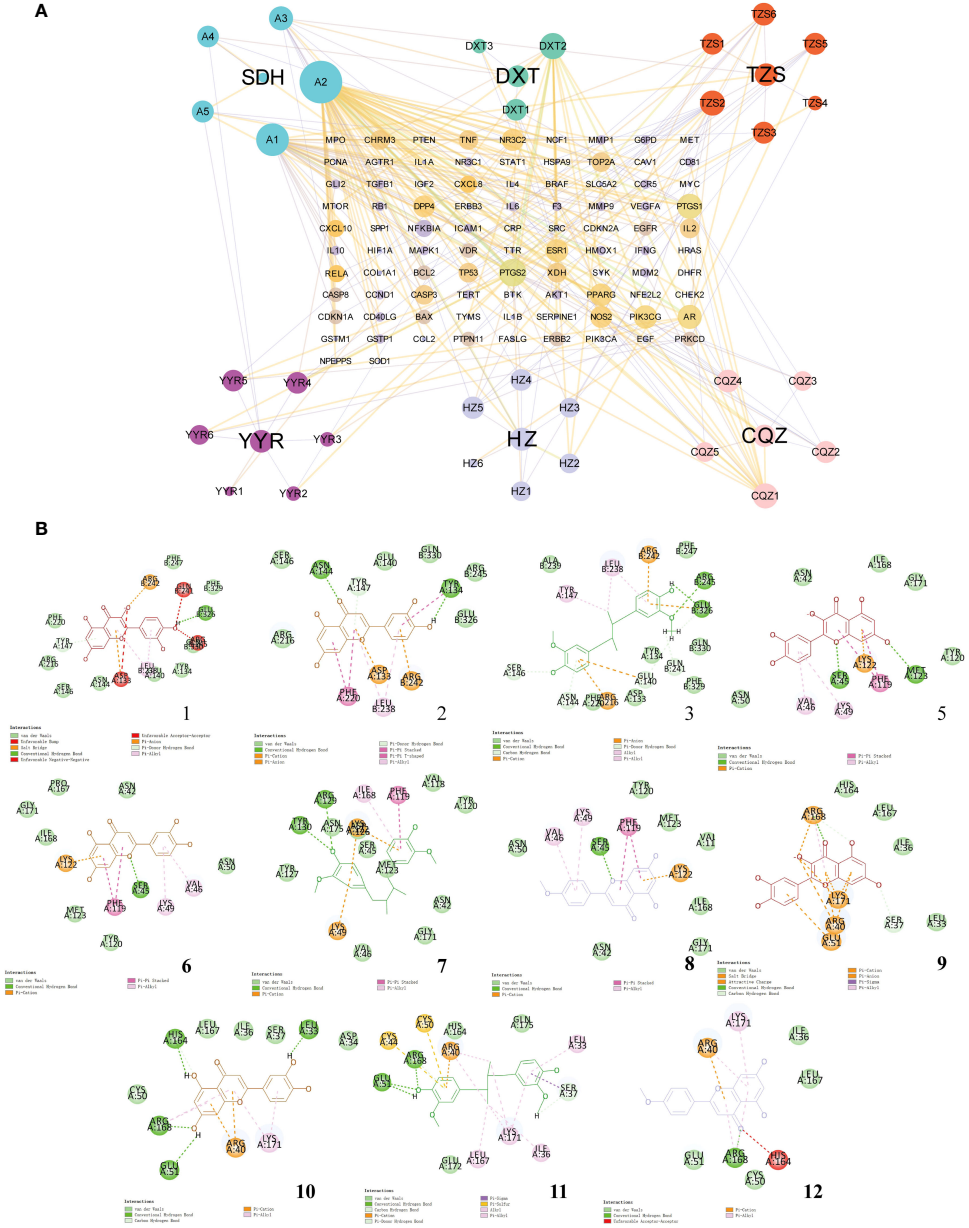
Network pharmacological analysis of the Tailin-II formula intervention for UTI. **(A)** Construction of the “Herb–Component–Target” network. **(B)** Molecular docking verification of natural compounds and core target proteins.

To gain deeper insights into the relationship between natural compounds and target genes, we constructed an “Herb-Component-Target” network was constructed using Cytoscape (**Figure 3A**). The node sizes in the network reflected the significance of the represented target or compound, with quercetin, luteolin, meso-dihydroguaiaretic acid, and acetin displaying high centrality throughout the study. Using the cytohubba plugin, the degree values were calculated to filter core genes. Applying this algorithm, we identified IL-1B, PTGS2, STAT1, AKT1, IL-6, RELA, EGF, CASP3, TNF, and NFKBIA were identified as genes with higher centrality in the network. Subsequently, the binding of key compounds to core targets was confirmed through analytical docking (**Figure 3B**). As components of the NF-κB signaling pathway, PTGS2, RELA, and IL-6 were selected as targets of interest. Details of molecular docking, including absolute energy, relative energy, and LibDock score, are presented in **Supplementary Table S2**. Higher docking scores suggest stronger binding between small-molecule compounds and target proteins.

### 3.3 TLF-II attenuates UPEC-induced bladder injury and bacterial burden

**Figure 4:**
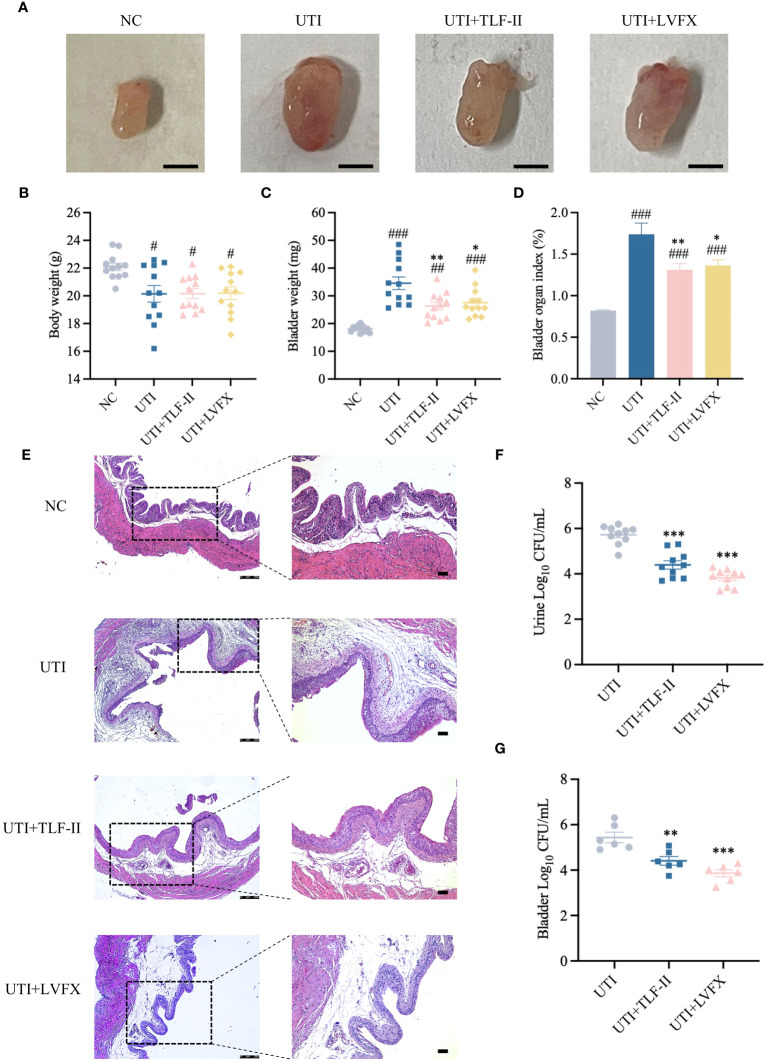
Protective effect of TLF-II on UTI-induced bladder injury. **(A)** Images of mouse bladders from each group (Scale bar = 3 mm, n=8). **(B–D)** Graphs showing body weight **(B)**, bladder weight **(C)**, and bladder organ index **(D)** (calculated as mouse bladder weight (mg)/body weight (g) × 100%, n=10). **(E)** HE staining of bladder tissue sections in each group (magnifications: ×50 and ×100, Scale bar = 75 μm, n = 4). **(F)** Quantification of bacterial load in urines (log10 fold CFU/mL) from mice in each group, n = 10. **(G)** Bacterial load in mouse bladders (log10 fold CFU/mL), n = 6. Data are represented as mean ± SEM. Significance is indicated as follows: #p < 0.05, ##p < 0.01, ###p < 0.001 vs. NC group; *p < 0.05, **p < 0.01, ***p < 0.001 vs. UTI group.

To evaluate TLF-II’s urethral protective effects, a UTI mouse model was established by transurethrally inoculating UPEC CFT073 into Balb/C mice. Bladder morphological and pathological changes in mice were observed following TLF-II and LVFX treatments, respectively. After successful modeling, UTI mice exhibited visibly congested and swollen bladders, inflammatory foci, and a significant increase in bladder weight. These symptoms were markedly alleviated after TLF-II intervention, mirroring the LVFX group (**Figure 4A**). To quantitatively assess bladder injury, the bladder organ index was calculated for each mouse. The results indicated a 1.32- and 1.28-fold increase in the bladder organ index of UTI mice relative to TLF-II and LVFX, respectively (**Figures 4B–D**), signifying TLF-II’s superior efficacy in attenuating UTI-induced bladder injury over LVFX. The histological examination further supported these findings. As depicted in **Figure 4E**, TLF-II treatment resulted in significant histopathological improvements, characterized by mild inflammatory cell infiltration, a reduced number of chemotactic epithelial layers, mild tissue edema, and mild interstitial fibrous tissue proliferation, aligning with the LVFX group’s outcomes. This finding confirms TLF-II’s ability to reduce tissue inflammatory responses triggered by UTI. Additionally, the effect of TLF-II on UPEC’s ability to cause acute infections was investigated by examining bacterial titers in the urine samples and bladder tissues of mice. The results indicated a 0.77-log-fold reduction in bacterial titers in the urine of TLF-II-treated mice compared with those of UTI mice (**Figure 4F**). A similar trend was observed in the bacterial load in the bladder tissues of these mice (**Figure 4G**). These findings suggest that TLF-II effectively inhibits bacterial growth during acute infections, comparable to LVFX, while also reducing the extent of bladder injury.

### 3.4 TLF-II attenuates UPEC-induced inflammation by regulating TLR4-NF-κB pathways

**Figure 5:**
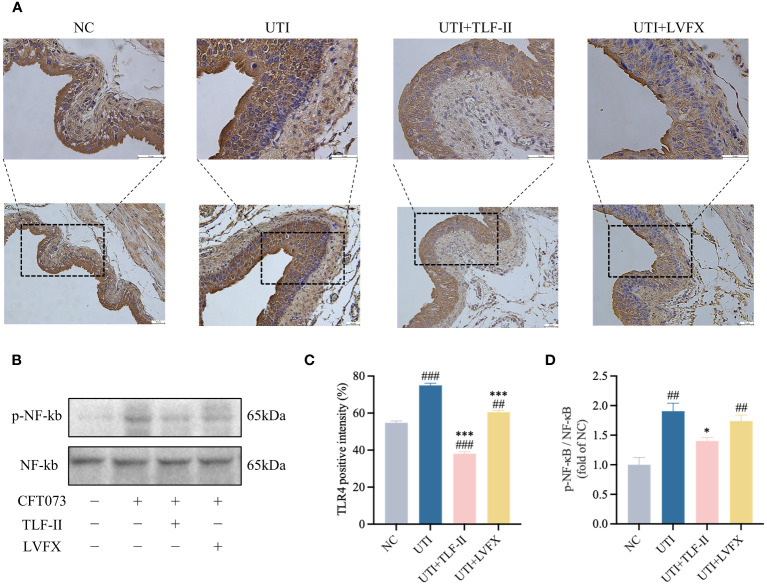
TLF-II affects the TLR4- NF-κB pathway. **(A)** Immunohistochemical staining of mouse bladders showing TLR4 immunodetection (brown) in BECs (Scale bar = 50 μm). **(B)** Western blot analysis of both phosphorylated and total NF-κB. **(C)** Quantitative analysis of the positive intensity of TLR4 in BECs. **(D)** Quantification of protein bands for phosphorylated NF-κB with total NF-κB as a control. Data are represented as mean ± SEM of three independent experiments. Significance is indicated as the p value, ##p < 0.01, ###p < 0.001 vs. NC group; *p < 0.05, ***p < 0.001 vs. UTI group.

**Figure 6:**
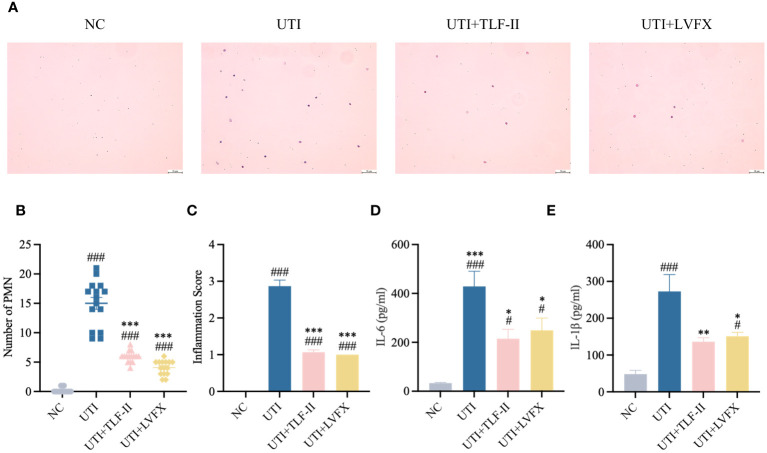
TLF-II inhibits UTI–induced inflammatory responses. **(A)** Urinary sediments from each group of mice were stained and analyzed. Representative urine cytology images from each group (Scale bar = 50 μm). **(B, C)** Graphs showing polymorphonuclear leukocyte counts **(B)** and urine inflammation scores **(C)** per mouse after different interventions. **(D, E)** Serum levels of IL-6 **(D)** and IL-1β **(E)** determined by ELISA. Data are represented as mean ± SEM of three independent experiments. Significance is indicated as the p value, #p < 0.05, ###p < 0.001 vs. NC group; *p<0.05, **p < 0.01, ***p <0.001 vs. UTI group.

During UPEC infection, the bladder can shed bacteria into the lumen by exfoliating infected BECs (Stemler et al., 2013). However, this shedding process triggers an inflammatory response and prompts the remaining urothelial cells to proliferate (Dalghi et al., 2020). To exclude the possibility that the lower bladder bacterial load observed in TLF-II-treated mice, as demonstrated above, was due to increased exfoliation, the inflammatory response in each group was assessed. TLR4 molecules on BECs recognize UPEC’s LPS, rapidly initiating the innate immune response and releasing inflammatory cytokines (Song et al., 2009). Therefore, the expression levels of TLR4 were initially compared among the groups. The results indicated a significant decrease in TLR4 expression after TLF-II intervention compared with that in the UTI group (**Figures 5A, C**). As NF-κB plays a pivotal role in TLR4 signaling, NF-κB activation post-TLF-II intervention was examined. The findings revealed lower levels of NF-κB phosphorylation in TLF-II-treated mice than in both UTI and LVFX mice (**Figures 5B, D**), implying that TLF-II can inhibit NF-κB activation and exert anti-inflammatory effects. Further investigation of PMN counts in the urine of mice (**Figure 6A**) revealed a decrease in the influx of inflammatory cells in the urine of TLF II-intervened mice compared to UTI mice (**Figures 6B, C**). This suggests that fewer immune cells were recruited and activated after TLF-II treatment. Additionally, pro-inflammatory cytokine levels in the blood of mice were compared, and the results showed significantly lower levels of IL-1β and IL-6 in TLF-II-treated mice than in UTI mice (**Figures 6D, E**). These data confirm the effectiveness of TLF-II in mitigating inflammation during UTI episodes, with its anti-inflammatory mechanism likely exerted through the modulation of the TLR4-NF-κB pathway.

### 3.5 TLF-II inhibits SCL20A1 to prevent UPEC from escaping from fusiform vesicles

**Figure 7:**
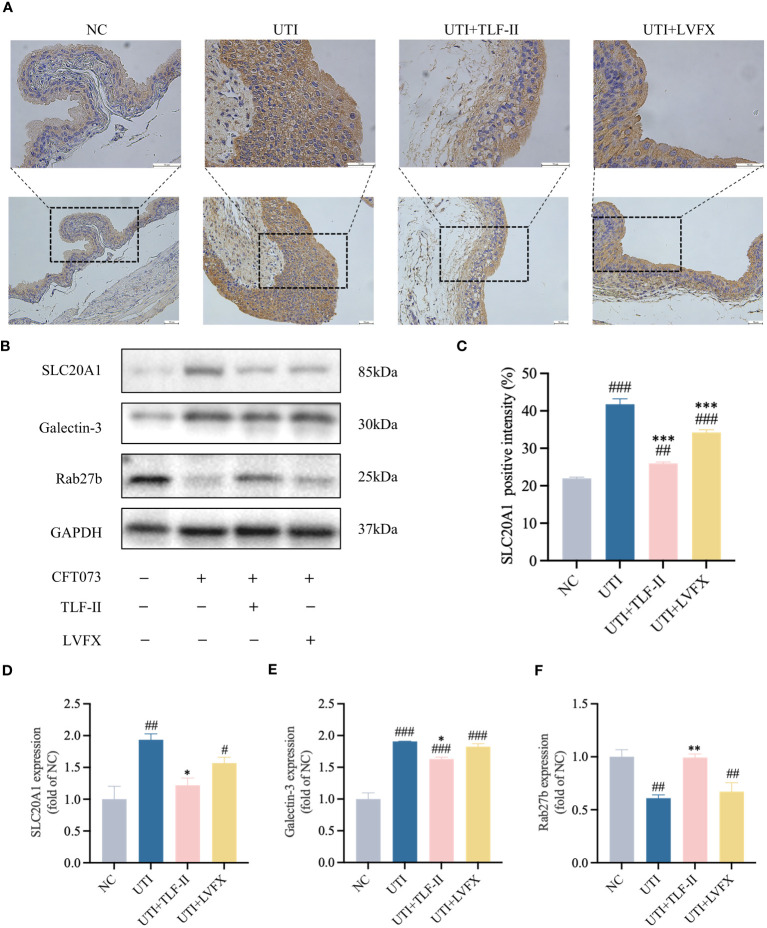
TLF-II protects fusiform vesicles and inhibits the occurrence of immune escape in bacteria. **(A)** Immunohistochemical staining of mouse bladders showing immunodetection of SLC20A1 (brown) in bladder epithelial cells (BECs; scale bar = 50 μm). **(B)** Western blot analysis of SLC20A1, Galectin-3, and Rab27b protein levels. **(C)** Quantitative analysis of the positive intensity of SLC20A1 in BECs. **(D–F)** Quantified protein bands of SLC20A1 **(D)**, Galectin-3 **(E)**, and Rab27b **(F)** with GAPDH as a control group. Data are represented as mean ± SEM of three independent experiments. Significance is indicated as the p value, #p < 0.05, ##p < 0.01, ###p < 0.001 vs. NC group; *p<0.05, **p < 0.01, ***p <0.001 vs. UTI group.

**Figure 8:**
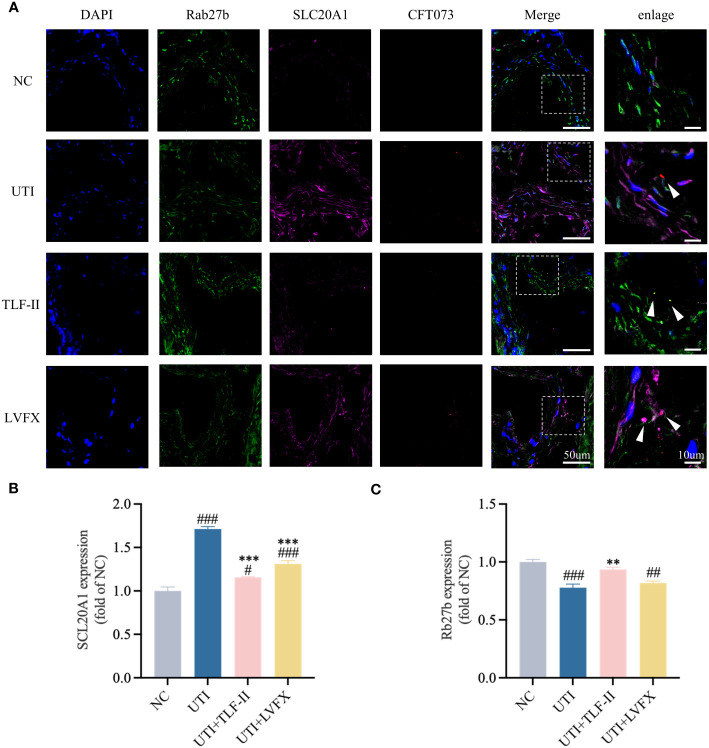
TLF-II reduces bacterial escape from fusiform vesicles into the cytoplasm. **(A)** Immunofluorescence staining of Rab27b, SLC20A1, UPEC-CFT073 and DAPI, along with co-localization of Rab27b, SLC20A1, and UPEC-CFT073, indicated by white arrows in the enlarged image. **(B, C)** Mean fluorescence intensity of Rab27b **(B)** and SLC20A1 **(C)** measured in randomly selected per view. Data are represented as mean ± SEM of three independent experiments. Significance is indicated as the p value, #p < 0.05, ##p < 0.01, ###p < 0.001 vs. NC group; **p < 0.01, ***p < 0.001 vs. UTI group.

SCL20A1 is reported to be present on the membrane of fusiform vesicles labeled by Rab27b in BECs of mouse bladders (Pang et al., 2022). It facilitates UPEC escape into the cytosol by reducing phosphate concentration in BCVs (Rieke et al., 2020; Pang et al., 2022). To investigate TLF-II’s ability to prevent UPEC from escaping from fusiform vesicles into the cytoplasm, we examined the effect of TLF-II on the expression of SLC20A1 and Rab27b. The results demonstrated decreased SLC20A1 expression levels after TLF-II intervention compared with that in the UTI and LVFX groups (**Figures 7A–D**), whereas Rab27b expression was elevated (**Figures 7B, F**). Confocal microscopy revealed significant co-localization of SLC20A1, Rab27b, and UPEC in UTI mice, with observable UPEC escape from the vesicles (**Figure 8A**). Conversely, TLF-II-treated mice exhibited increased co-localization of UPEC with Rab27b and reduced bacterial escape (**Figure 8A**), and the levels of SLC20A1 and Rab27b were consistent with the results of western blotting (**Figures 8B, C**). Galectin-3 binds to β-galactose-containing glycoconjugates in lysed fusiform vesicles, reflecting vesicle lysis (Paz et al., 2010). The findings indicated that TLF-II reduced Galectin-3 expression in mouse bladder BECs (**Figures 7B, E**), suggesting decreased vesicle lysis after TLF-II intervention. These results collectively suggest that TLF-II protects fusiform vesicles by inhibiting SLC20A1, thus trapping UPEC in BCVs and reducing their escape.

## 4 Discussion

UPEC stands as the primary causative agent of UTI, responsible for 75%-95% of all cases. Currently, antibiotics remain the primary treatment for UTI, but excessive use of antibiotics can lead to antibiotic resistance, microbiota disruption (Cannon, 2014; Benito-Villalvilla et al., 2017), and potential side effects such as liver and kidney toxicity (Reardon, 2014). Consequently, there is an urgent demand for innovative UTI treatment approaches. Some commonly used herbs possess diuretic, antibacterial, anti-inflammatory, immune-boosting, and pain-relieving properties, offering a biological basis for the potential of CHM in UTI treatment (Liu et al., 2019; Kranz et al., 2022; Li et al., 2022).

**Figure 9:**
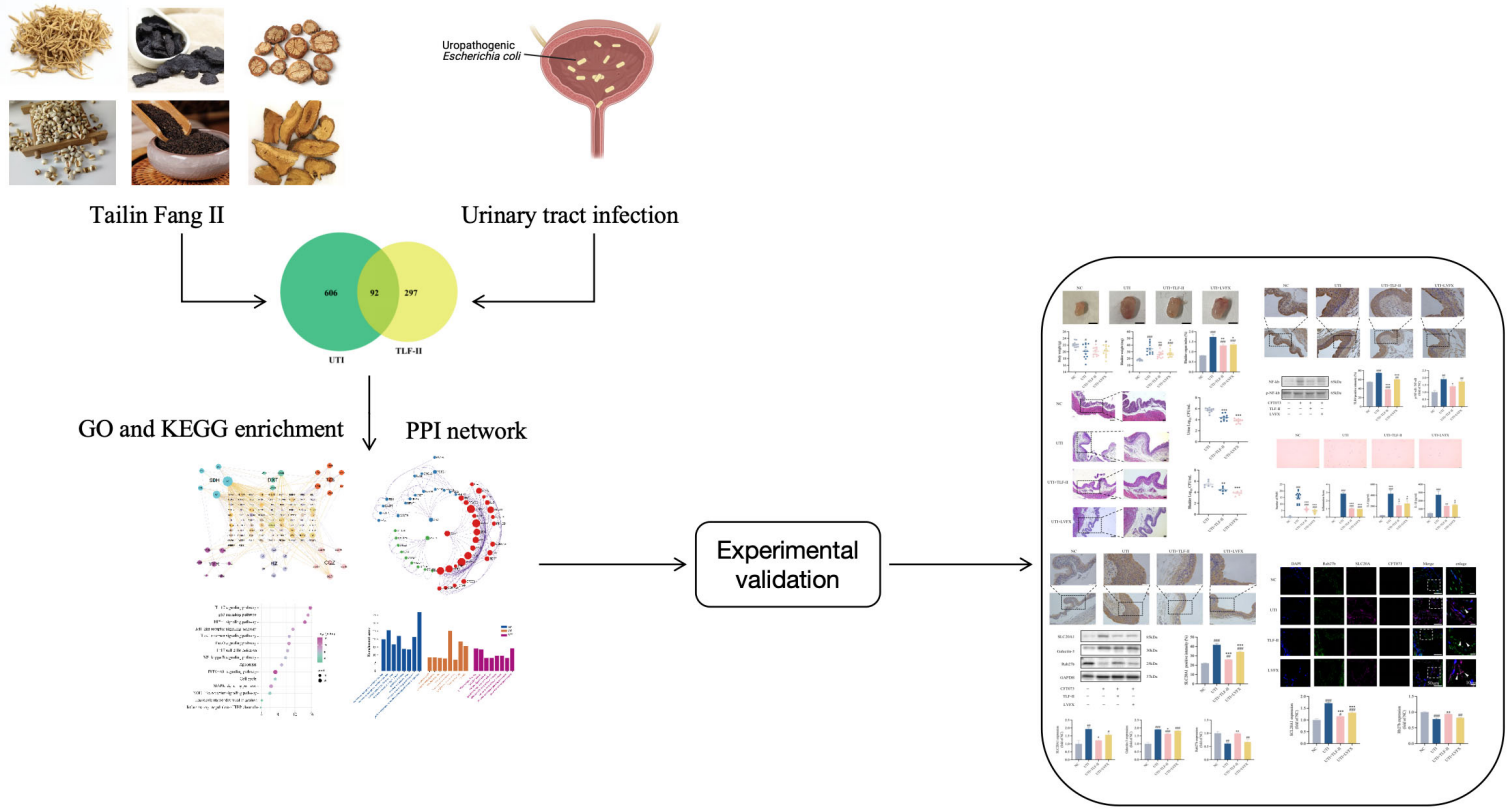
Flowchart outlining all procedures in the study.

TLF-II, an empirical formula for UTI treatment frequently employed in clinical practice, and it has proven effective in alleviating clinical symptoms. However, the basic research on TLF-II is lacking, and its mechanism is unclear. In this study (**Figure 9**), based on the results of network pharmacology analysis, we screened several pathways related to natural immunity and inflammation, among which TLR4-NFκB was of interest to us. In animal experiments, the Balb/C mouse model is widely used in studies of UTI caused by uropathogenic Escherichia coli (Shea et al., 2020; Habibi et al., 2021; Dickson et al., 2023). It has been shown to establish persistent intracellular bacterial communities in their urinary epithelial cells (Chockalingam et al., 2019). Therefore, Balb/C mice were used throughout the experiments. UTI was induced by inoculating the urethra of Balb/C mice with an infectious dose of UPEC, and modeling success was determined by detecting UPEC CFT073 quantities ≥10^4^ CFU/mL in urine. Levofloxacin was chosen as a positive control group. Levofloxacin is a frequently prescribed antibiotic for treating urinary tract infections. Many studies have used levofloxacin as a control drug for urinary tract infections Wagenlehner et al., 2015; Kaye et al., 2018; Mo et al., 2022), consistently demonstrating its superior and stable efficacy compared to other antibiotics (Li et al., 2021; Xue et al., 2021; Popa et al., 2015; Rouphael et al., 2023). Given its widespread use in clinical settings, selecting levofloxacin as a control drug may provide a more representative sample.

After UPEC infects the urinary tract, the type-1 fimbriae on its surface facilitate bacterial binding to uroplakin 1a molecules on superficial BECs, leading to bacterial adhesion. The urinary tract relies on its innate immune defense system as a crucial line of defense against infection. TLR4, expressed in BECs, primarily recognizes LPS on the surface of pathogenic bacteria. TLR4-LPS binding results in NF-κB activation and the secretion of pro-inflammatory cytokines and chemokines, playing a vital role in bacterial clearance and inflammatory response (Jones-Freeman et al., 2021). Interleukins are a crucial member of the cytokine family that directly promote the growth, activation, adhesion, differentiation, migration, proliferation, and maturation of immune cells. And subsequently, they should play significant roles in the body’s proinflammatory and anti-inflammatory responses through interactions with a variety of receptors (Behzadi et al., 2022). IL-6 and IL-1β are commonly utilized as markers to detect inflammation levels in UTIs, and their increased levels indicate a higher severity of UTI (Ching et al., 2018; Huang et al., 2023).

In our study, we utilized the UPEC-induced UTI mouse model to explore the mechanism of TLF-II against UTI by modulating innate immunity. As Balb/C mice are naturally resistant to prolonged UTI (Murray et al., 2021), we sacrificed the mice 72 h post-modeling to observe the preventive and curative effects of TLF-II on cystitis. Our results suggest that TLF-II effectively reduces bacterial load and bladder injury, and potentially modulates the innate immune response through regulating the TLR4-NFκB pathway and inhibiting inflammatory factors and immunocyte production.

Rab27b, a small GTPase of the Rab family, is highly expressed in BECs and plays a crucial role in the targeted delivery of fusiform vesicles to the apical plasma membrane of BECs, serving as a specific marker for these vesicles (Song et al., 2009; Pang et al., 2022; Chen et al., 2003). UPEC successfully colonizes superficial BECs by breaching the mucosal barrier and takes refuge in fusiform vesicles within infected BECs (Song et al., 2009). SLC20A1, expressed in the bladder, is primarily responsible for transporting phosphate into the host cell cytoplasm. Isolation of BCVs from 5637 cells using magnetic nanoparticle-tagged bacteria revealed the presence of SLC20A1 in these vesicles (Pang et al., 2022). SLC20A1 expression rises upon UPEC infection, with UPEC sensing the low phosphate concentration within BCVs activating its phospholipase pldA. pldA, located on UPEC’s outer membrane, in its monomeric form, becomes active upon dimerization and interaction with its phospholipid substrate (Snijder et al., 1999). As a key UPEC enzyme, pldA has been shown to disrupt the fusiform vesicle membrane, facilitating UPEC’s escape from fusiform vesicles, evading host immune defenses, and allowing UPEC to enter the cytoplasm to form IBCs (Pang et al., 2022). Our data suggest that TLF-II effectively reduces bacterial escape may through inhibiting SLC20A1 expression and protecting Rab27b-labeled vesicles, lowering the risk of recurrent infection.

Overall, the present study results indicate that TLF-II treatment effectively reduced bladder injury and bacterial load in mice, decreased the levels of TLR4 and NF-κB, as well as the expression of inflammatory factors IL-1β and IL-6, and suppressed UTI-induced inflammation. Statistical results confirmed this finding, with the TLF-II group exhibiting statistically significant differences in the levels of each inflammatory factor compared with the UTI group (p<0.05). Importantly, TLF-II also diminished bacterial escape from fusiform vesicles into the cytoplasm, a key mechanism for inhibiting IBCs formation and thus reducing UTI recurrence. Statistical analysis showed significant increases in SLC20A1 and Galectin-3 levels and a decrease in Rab27b level in the TLF-II group compared to the UTI and LVFX groups, and the difference was statistically significant (p<0.05).

Interestingly, recent studies have unveiled a relationship between SLC20A1 and NF-κB-dependent inflammation, with SLC20A1 mRNA upregulation during NF-κB pathway activation. Reducing SLC20A1 led to decreased IκB degradation, p65 nuclear translocation, and a drop in inflammatory factors such as IL-6 (Koumakis et al., 2019). Silencing NF-κB p65 in 5637 cells infected with UPEC resulted in significantly lower SCL20A1 expression compared with unsilenced cells (Pang et al., 2022). These findings align with the results of our study. It provides a novel insight that UPEC may induce host cell SCL20A1 expression through NF-κB in BECs. Nevertheless, the precise nature of their relationship requires further elucidation.

Existing literature primarily focuses on TCM’s bacterial elimination and inhibition of UPEC biofilm formation for UTI treatment (Liu et al., 2017; Flower et al., 2019; Chen et al., 2023). Recent study, however, delves into a pivotal molecular mechanism in UPEC’s pathogenic process—the escape of UPEC from fusiform vesicles into the cytoplasm to form IBCs (Pang et al., 2022). This opens a novel avenue for UTI treatment. To the best of our knowledge, this is the first study to investigate the potential of drugs in preventing and treating UTI by inhibiting the immune escape of UPEC from fusiform vesicles. Our study aims to establish a theoretical foundation for the clinical management of UTI. However, it has been reported that 15% of intracellular UPEC CI5 virulent strains are not present in BCVs of human BECs line 5637, and IBCs formation is rarely observed in these cells (Eto et al., 2006; Bishop et al., 2007), so we have not conducted *in vitro* experiments on UPEC infection. Furthermore, the active ingredients of TLF-II, which play significant roles against UTI, were not studied. Additionally, the modeling time for mice needs to be extended to evaluate the recurrence rate of UTI. In the future, we will further explore the active drug monomers that play a role in TLF-II and extend our observation time to further inform the prevention and treatment of RUTI from the perspective of bacterial escape in combination with *in vivo* and *in vitro* experiments.

## 5 Conclusion

Our present study demonstrated that TLF-II suppressed UPEC-induced inflammation and bladder injury by regulating the TLR4-NF-κB pathway activation and the innate immune response. Most notably, TLF-II hindered bacterial escape from fusiform vesicles into the cytoplasm, which is a key mechanism in treating UTI and reducing recurrence. These findings establish the theoretical foundation and biological background for TLF-II against UTI.

## Data availability statement

The raw data supporting the conclusions of this article will be made available by the authors, without undue reservation.

## Ethics statement

The studies involving humans were approved by Shanghai Hospital of Traditional Chinese Medicine Ethics Committee. The studies were conducted in accordance with the local legislation and institutional requirements. The participants provided their written informed consent to participate in this study. The animal study was approved by Shanghai Hospital of Traditional Chinese Medicine Ethics Committee. The study was conducted in accordance with the local legislation and institutional requirements.

## Author contributions

Z-PL: Writing – original draft, Conceptualization, Formal analysis, Investigation, Methodology, Visualization. JL: Writing – original draft, Formal analysis, Software. T-LL: Writing – review & editing, Investigation, Resources. Z-YS: Writing – review & editing, Investigation. X-ZG: Writing – review & editing, Conceptualization, Funding acquisition, Methodology, Supervision, Validation.
