# Supplementary figures and images for: Uropathogenic Escherichia coli infection: innate immune disorder, bladder damage, and Tailin Fang II
Source: Front Cell Infect Microbiol. 2024 Apr 4;14:1322119. doi: 10.3389/fcimb.2024.1322119 (PMC11024302; doi:10.3389/fcimb.2024.1322119)

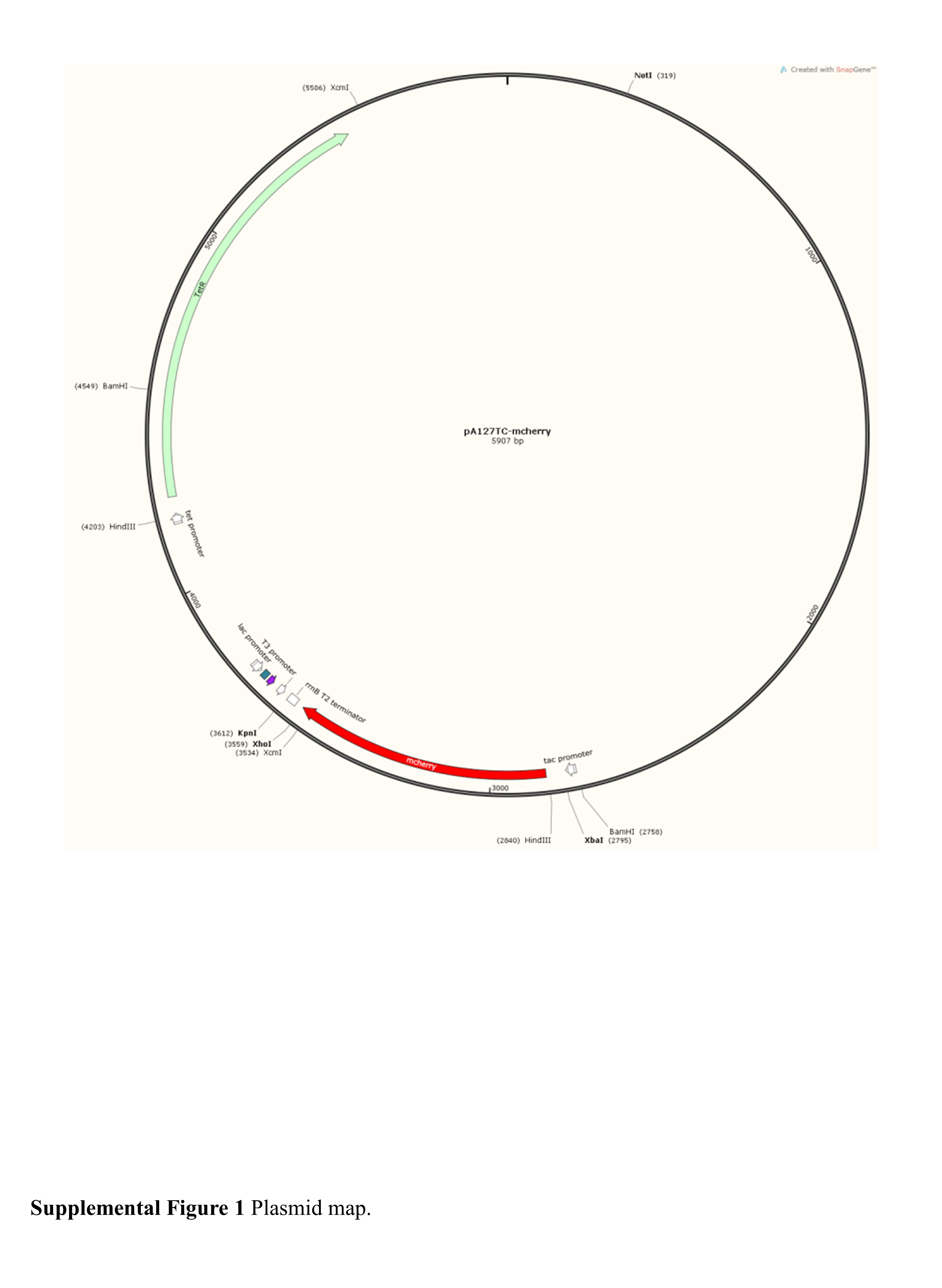

Supplement: Supplementary file 1 [file Image_1.tif]
