# Supplementary material for: Uropathogenic Escherichia coli infection: innate immune disorder, bladder damage, and Tailin Fang II
Source: Front Cell Infect Microbiol. 2024 Apr 4;14:1322119. doi: 10.3389/fcimb.2024.1322119 (PMC11024302; doi:10.3389/fcimb.2024.1322119)
Supplement: Supplementary file 4 [file Table_1.docx]

| **Name** | **Natural compounds** | **Molecular weight** | **Oral bioavailability** | **Drug-likeness** |
| --- | --- | --- | --- | --- |
| A1 | luteolin | 286.25 | 36.16 | 0.25 |
| A2 | quercetin | 302.25 | 46.43 | 0.28 |
| A3 | beta-sitosterol | 414.79 | 36.91 | 0.75 |
| A4 | sitosterol | 414.79 | 36.91 | 0.75 |
| A5 | Stigmasterol | 412.77 | 43.83 | 0.76 |
| CQZ1 | 3-Epioleanolic Acid | 456.78 | 32.03 | 0.76 |
| CQZ2 | Dinatin | 300.28 | 30.97 | 0.27 |
| CQZ3 | Dihydrotricetin | 304.27 | 58.12 | 0.28 |
| CQZ4 | Hypolaetin | 302.25 | 33.24 | 0.28 |
| CQZ5 | plantaginin_qt | 288.27 | 54.04 | 0.24 |
| DXT1 | (-)-catechin | 290.29 | 49.68 | 0.24 |
| DXT2 | meso-dihydroguaiaretic acid | 330.46 | 31.32 | 0.26 |
| DXT3 | 2-(4-hydroxyphenyl)ethyl (E)-3-(4-hydroxyphenyl)prop-2-enoate | 284.33 | 93.36 | 0.21 |
| HZ1 | (+)-catechin | 290.29 | 54.83 | 0.24 |
| HZ2 | Physciondiglucoside | 608.6 | 41.65 | 0.63 |
| HZ3 | rhein | 284.23 | 47.07 | 0.28 |
| HZ4 | 6,8-Dihydroxy-7-methoxyxanthone | 258.24 | 35.83 | 0.21 |
| HZ5 | Physovenine | 262.34 | 106.21 | 0.19 |
| HZ6 | Picralinal | 366.45 | 58.01 | 0.75 |
| TZS1 | Supraene | 410.8 | 33.55 | 0.42 |
| TZS2 | acacetin | 284.28 | 34.97 | 0.24 |
| TZS3 | Linarin | 592.6 | 39.84 | 0.71 |
| TZS4 | 1-Monolinolein | 354.59 | 37.18 | 0.3 |
| TZS5 | Taraxerol | 426.8 | 38.4 | 0.77 |
| TZS6 | Schottenol | 414.79 | 37.42 | 0.75 |
| YYR1 | CLR | 386.73 | 37.87 | 0.68 |
| YYR2 | Sitosterol alpha1 | 426.8 | 43.28 | 0.78 |
| YYR3 | Mandenol | 308.56 | 42 | 0.19 |
| YYR4 | Hydrosqualene | 410.8 | 33.55 | 0.42 |
| YYR5 | Coixenolide | 591.08 | 32.4 | 0.43 |
| YYR6 | 2-Monoolein | 356.61 | 34.23 | 0.29 |

**Supplementary Table 1.** Pharmacokinetic properties of natural compounds in the "herb-component-target" network.
