# Supplementary material for: Uropathogenic Escherichia coli infection: innate immune disorder, bladder damage, and Tailin Fang II
Source: Front Cell Infect Microbiol. 2024 Apr 4;14:1322119. doi: 10.3389/fcimb.2024.1322119 (PMC11024302; doi:10.3389/fcimb.2024.1322119)
Supplement: Supplementary file 5 [file Table_2.docx]

| **ID** | **Targets** |  | **Absolute energy** | **Relative energy** | **LibDock score** |
| --- | --- | --- | --- | --- | --- |
| 1 | PTGS2 | quercetin | 37.0259 | 0 | 82.0165 |
| 2 | PTGS2 | luteolin | 34.4952 | 0.0431269 | 73.8028 |
| 3 | PTGS2 | meso-dihydroguaiaretic acid | 63.0639 | 14.5206 | 92.1818 |
| 4 | PTGS2 |  | N | N | N |
| 5 | RELA | quercetin | 37.0287 | 0.00281372 | 72.1391 |
| 6 | RELA | luteolin | 34.4952 | 0.0431269 | 73.6889 |
| 7 | RELA | meso-dihydroguaiaretic acid | 55.8801 | 7.33678 | 94.12 |
| 8 | RELA | acacetin | 45.2558 | 0 | 78.0193 |
| 9 | IL-6 | quercetin | 37.0287 | 0.00281372 | 71.4489 |
| 10 | IL-6 | luteolin | 34.4952 | 0.0431269 | 85.6855 |
| 11 | IL-6 | meso-dihydroguaiaretic acid | 58.5959 | 10.0526 | 101.35 |
| 12 | IL-6 | acacetin | 45.2558 | 0 | 75.225 |

**Supplementary Table 2.** Molecular docking validation results.
